# Three-dimensional genome landscape comprehensively reveals patterns of spatial gene regulation in papillary and anaplastic thyroid cancers: a study using representative cell lines for each cancer type

**DOI:** 10.1186/s11658-022-00409-6

**Published:** 2023-01-06

**Authors:** Linlin Zhang, Miaomiao Xu, Wanchun Zhang, Chuanying Zhu, Zhilei Cui, Hongliang Fu, Yufei Ma, Shuo Huang, Jian Cui, Sheng Liang, Lei Huang, Hui Wang

**Affiliations:** 1 Department of Nuclear Medicine, Xinhua Hospital Affiliated to Shanghai Jiao Tong University School of Medicine, Shanghai, 200092 China; 2 Department of Nuclear Medicine, Shanxi Bethune Hospital (Shanxi Academy of Medical Sciences), Taiyuan, 03003 China; 3 Department of Oncology, Xin Hua Hospital Affiliated to Shanghai Jiaotong University School of Medicine, Shanghai, 200092 China; 4 Department of Respiratory Medicine, XinHua Hospital Affiliated to Shanghai Jiao Tong University School of Medicine, Shanghai, 200092 China; 5 BioGenius Bioinformatics Institute, Shanghai, 200050 People’s Republic of China; 6 Department of Oncology, Ruijin Hospital, Shanghai Jiao Tong University School of Medicine, Shanghai, 200025 China; 7 Medical Center on Aging of Ruijin Hospital, Shanghai Jiao Tong University School of Medicine, Shanghai, 200025 China

**Keywords:** Papillary thyroid cancer, Anaplastic thyroid cancer, Genomic heterogeneity, Topologically associating domains (TADs), High-throughput chromosome conformation capture (Hi-C), A/B compartment switches, Somatic hotspot mutations, Structural variations, Copy number variations, RNA sequencing, Whole-genome sequencing

## Abstract

**Background:**

Spatial chromatin structure is intricately linked with somatic aberrations, and somatic mutations of various cancer-related genes, termed co-mutations (CoMuts), occur in certain patterns during cancer initiation and progression. The functional mechanisms underlying these genetic events remain largely unclear in thyroid cancer (TC). With discrepant differentiation, papillary thyroid cancer (PTC) and anaplastic thyroid cancer (ATC) differ greatly in characteristics and prognosis. We aimed to reveal the spatial gene alterations and regulations between the two TC subtypes.

**Methods:**

We systematically investigated and compared the spatial co-mutations between ATC (8305C), PTC (BCPAP and TPC-1), and normal thyroid cells (Nthy-ori-3–1). We constructed a framework integrating whole-genome sequencing (WGS), high-throughput chromosome conformation capture (Hi-C), and transcriptome sequencing, to systematically detect the associations between the somatic co-mutations of cancer-related genes, structural variations (SVs), copy number variations (CNVs), and high-order chromatin conformation.

**Results:**

Spatial co-mutation hotspots were enriched around topologically associating domains (TADs) in TC. A common set of 227 boundaries were identified in both ATC and PTC, with significant overlaps between them. The spatial proximities of the co-mutated gene pairs in the two TC types were significantly greater than in the gene-level and overall backgrounds, and ATC cells had higher TAD contact frequency with CoMuts > 10 compared with PTC cells. Compared with normal thyroid cells, in ATC the number of the created novel three-dimensional chromatin structural domains increased by 10%, and the number of shifted TADs decreased by 7%. We found five TAD blocks with CoMut genes/events specific to ATC with certain mutations in genes including *MAST-NSUN4*, *AM129B*/*TRUB2*, *COL5A1*/*PPP1R26*, *PPP1R26*/*GPSM1*/*CCDC183*, and *PRAC2*/*DLX4*. For the majority of ATC and PTC cells, the HOXA10 and HIF2α signals close to the transcription start sites of CoMut genes within TADs were significantly stronger than those at the background. CNV breakpoints significantly overlapped with TAD boundaries in both TC subtypes. ATCs had more CNV losses overlapping with TAD boundaries, and noncoding SVs involved in intrachromosomal SVs, amplified inversions, and tandem duplication differed between ATC and PTC. TADs with short range were more abundant in ATC than PTC. More switches of A/B compartment types existed in ATC cells compared with PTC. Gene expression was significantly synchronized, and orchestrated by complex epigenetics and regulatory elements.

**Conclusion:**

Chromatin interactions and gene alterations and regulations are largely heterogeneous in TC. CNVs and complex SVs may function in the TC genome by interplaying with TADs, and are largely different between ATC and PTC. Complexity of TC genomes, which are highly organized by 3D genome-wide interactions mediating mutational and structural variations and gene activation, may have been largely underappreciated. Our comprehensive analysis may provide key evidence and targets for more customized diagnosis and treatment of TC.

**Supplementary Information:**

The online version contains supplementary material available at 10.1186/s11658-022-00409-6.

## Background

Thyroid cancer (TC) remains one of the most common malignancies of the endocrine system, and is the fifth most common cancer in women [1, 2]. In 2020, more than 586,000 new cases of TC and about 44,000 new deaths associated with TC were estimated [3, 4]. Overall, the incidence of TC is increasing, which is primarily due to an increase in the incidence of papillary TC (PTC), a subtype of differentiated TC [5]. Anaplastic TC (ATC), though less common, is the most aggressive form of TC with a median survival time of 3–5 months [6, 7]. With discrepant differentiation, ATC and PTC differ greatly in characteristics and prognosis.

It is well known that genomic aberrations are related to cancer initiation and progression along with incidences of critical events, including chromosome translocations and arrangements, single-nucleotide variants (SNVs), and copy number variations (CNVs) [8, 9]. Both ATC and PTC harbor a variety of genetic alterations, including mutations of *BRAF (V600E)*, *TERT* promoter, *TP53*, and *RAS* [10, 11]. A high proportion of TCs harbor mutations in the MAPK pathway, which has become a focal point for therapeutic intervention in TC [12]. *RAS*-mutated and *BRAF*-mutated poorly differentiated TCs (PDTCs) are characterized by profound undifferentiation, genomic complexity, and heavy mutation burden. Additionally, several specific CNVs are associated with poor prognosis [13]. Co-mutations of various somatic cancer-related genes are prevalent; however, the mechanisms underlying these genetic events in TC remain to be clarified. Despite recent evidence on various genomic aberrations reported for TC, no studies describing the interactions and correlations between different variation subtypes have been identified.

While the mutational landscape of ATC may somewhat resemble that of PTC, the clinical behavior of ATC and PTC is radically discrepant, which may be partly explained by the genetic differences. As the most frequent type of human thyroid carcinoma, PTC often has *BRAF (V600E)* point mutations, which could be a key target for the treatment of and an important marker for the diagnosis of most invasive PTCs [14, 15]. Most dedifferentiated, aggressive ATC has not only *RET* and *PAX8/PPARγ* rearrangements, but also *TP53* mutation [16, 17]. It has also been hypothesized that ATC derives from PTC through the progressive acquisition of a discrete number of genomic alterations. PTC-derived ATCs are characterized by *BRAF* and *TERT* promoter mutations, which occur prior to anaplastic transformation, and ATCs harboring *TERT* promoter mutation are at higher risk for anaplastic transformation [18]. A previous study further showed that ATC diverges from PTC early in tumor development and that both tumor types evolve independently [19]. Differences in genomic alterations between ATC and PTC need to be further revealed.

Chromosome compaction and organization are an evolutionarily conservative characteristic, during which large genomes are condensed into the three-dimensional (3D) space of the nucleus, so as to maintain the functional capacity to interplay with the machinery regulating genes. This promotes the fine-tuning expression of genes through regulating the contacts among located *cis*-regulatory elements [20]. Chromatin topology may exert vital functions in bringing enhancers into space that is proximal to their target genes. The genome could be divided into topologically associating domains (TADs) [21], and TADs are contiguous genomic segments with interactions more frequently within than between them [22–24]. TADs are regarded as functional domains since they include the regulation elements for the genes contained within the same domain [25]. Disrupting domain boundaries could lead to ectopic interplays between neighboring domains, which can influence gene regulation. Since chromatin can be organized into contact domains or TADs that are hundreds of kilobases (kb) in size, the chromosome conformation capture techniques (3C) is a popular method to elucidate the spatial organization of chromatin involved in the long range of gene regulation and epigenomic changes in cancers [26–29]. Due to advantages of this technique, diverse 3C-based methods have been developed during the past decade [27]. This has allowed investigators to study these somatic genome alterations regarding 3D genome-wide chromatin conformation.

Previous research has suggested strong associations between spatial proximity and chromosome rearrangements, and reported that the deregulated genes lay near the spatial proximity of translocation, suggesting their role in the development of ATC and PTC [30–34]. However, it remains largely obscure whether spatial chromatin structure is involved also in somatic aberrations.

To address the above issues, we compared the spatial co-mutations between ATC and PTC cells via whole-genome sequencing (WGS) and high-throughput chromosome conformation capture (Hi-C). We compared spatial proximity between genes that are co-mutated and those that are not co-mutated. Moreover, we characterized the conservation of the flanking sequences of the co-mutation signatures, and the disruption of signaling pathways by these driver mutations.

## Methods and materials

### Cell lines and cell culture

Normal human thyroid follicular epithelial cell line (Nthy-ori-3-1, cat. no. ZQ0874) and human TC cell lines (8305C for ATC, cat. no. ZQ0305 and BCPAP for PTC, cat. no. ZQ0304) were all purchased from Zhong Qiao Xin Zhou Biotechnology Co., Ltd. (Shanghai, China; http://fmgbio.bioon.com.cn). The PTC cell line TPC-1 (cat. no. FH1039) was purchased from Shanghai Fuheng Biotechnology Co., Ltd. (Shanghai, China; www.fudancell.com). Nthy-ori-3-1 cells were cultured in high-glucose DMEM (Sigma) containing 10% FBS (Gibco BRL) and 1% penicillin and streptomycin (PS). 8305C cells were cultured in the DMEM medium that was supplemented with 1% GlutaMAX, 10% FBS, and 1 mL nonessential amino acids (NEAA; 100×). BCPAP cells were cultured in the RPMI medium 1640 (Gibco BRL) that contained 1% PS (Gibco BRL), 10% FBS, 1% NEAA (Invitrogen), and 1% GlutaMAX (Invitrogen). TPC-1 cells were cultured in DMEM medium that contained 10% FBS (Gibco BRL) and 1% penicillin and streptomycin. All kinds of cell lines grew in the humidified atmosphere containing 5% CO_2_ at 37 °C. Further information, including catalog number, details, and availability of cell lines, is provided in Additional file 1: Table S1.

### Preparation of high-throughput chromosome conformation capture (Hi-C) library

The preparation of Hi-C chromatin conformation assay library, which comprises permeabilization of cells, fixation of chromatin, DpnII digestion, biotin labeling, ligation, and reversal of crosslink, was done as previously described by van Berkum et al. [35] with some minor modifications: (1) Before biotin labeling, we incubated samples at 65 °C for 25 min with sodium dodecyl sulfate (SDS), and subsequently quenched them using Triton-X; we made up both reagents to a final concentration of 1.3%. (2) Following proteinase-K digestion of the sample after ligation, we digested RNA by adding 40 μg/ml RNAse-A for 1 h at 37 °C. (3) The final concentration of biotinylated dCTP during labeling was changed to 0.025 mM. (4) Following biotin pulldown, DNA was fragmented and peaked at 500 bp. We did not perform selection of fragment size.

TADs represent a large-cell-type-invariant characteristic of genomic organization. We generated a common set of boundaries among discrepant types of cells. The high-resolution chromosome conformation datasets from three human-derived cell lines that represent two distinct TC subtypes (ATC and PTC) were used to identify boundaries of TADs in discrepant TC subtypes. We observed boundaries of TADs from 50-kb-binned Hi-C data for every cell type with a *z*-score for interaction. A score (signal of TAD) was calculated for every bin using this method, for the interactions with the nearby loci on average for a 50-kb genome window. Boundaries were determined to be regions that have local insulation minima along the diagonal of the Hi-C matrix.

### Hi-C contact analyses

A Hi-C contact matrix was generated following steps including mapping the Hi-C reads to the reference genome; filtering the aligned reads to create a contact matrix; filtering matrix bins with zero or low read coverage; and lastly removing biases from the contact Hi-C matrices. Once the Hi-C matrix was built, matrices at restriction fragment resolution could be created by providing a file containing the restriction sites, where the restriction sequence GATC was recognized by the DpnII restriction enzyme. We normalized the intrachromosomal 25-kb resolution using the Ruiz and Knight algorithm. The TAD signal was determined by moving a window across the diagonal of the Hi-C matrix, and the sum of the interplay for a given bin of up to 2-Mb flanking regions where log_2_ of the observed bin to the mean of the interplay values was inside the given 2-Mb window. The insulation score calculation method was used to identify boundaries of TADs [36].

The boundary regions were converted into binary bins per genome to compare the significance of overlaps between boundaries of TADs in various cell lines. We identified common boundaries of TADs for all three cell types that occurred within 50 kb in the genome range or 2 Hi-C bins. We applied this bootstrapping method to calculate the significance of the overlaps between boundaries of TADs in the TC cell lines.

### Whole-genome sequencing (WGS)

We performed preparation of library and sequencing using the BioGenius Genomics Platform following manufacturers’ instructions. We processed all cell lines using the same protocols for preparation of library and sequencing [37–39]. We prepared WGS libraries from 1 μg DNA using the Illumina TruSeq PCR-free DNA sample preparation kits with an average insert size of 350 bp. We did WGS clustering using cBot, and performed paired-end sequencing with 150-bp read length on Illumina HiSeqX (HiSeq Control Software Version 3.3.39/RTA 2.7.1) with sequencing chemistry (v2.5). The depth of coverage for each of the four analyzed cell lines was: PTC cell line (TPC-1), ~ 39.99 X with 99.98% of 119.969 Gb reads mapped on human genome hg19; PTC cell line (BCPAP), ~ 34.26 X with 99.81% of 102.7733Gb reads mapped on human genome hg19; ATC cell line (8305C), ~ 35.43 X with 97.47% of 106.2876Gb reads mapped on human genome hg19; normal thyroid cell line (Nthy-ori-3-1), ~ 31.61 X with 95.62% of 94.82678Gb reads mapped on human genome hg19.

### Analysis of structural alterations and copy number variations (CNVs)

Following the GATK4 guidelines, we aligned raw reads to the human reference genome GRCh37 (human_g1k_v37.fasta) using speedseq. SAMtools (v0.1.19) was used to sort and index the resulting alignments (BAM). Duplicates were removed via Picard. Somatic SNVs were identified by Mutect 2. DELLY and Lumpy algorithms were run to identify consensus structural variations (SVs) for cell lines. We included SV break-ends reported by two different callers for analysis. We obtained the consensus SV calls and annotations of every variation (inversions, duplications, deletions, and complex rearrangements). CNVs might be another confounding factor for the fold changes of gene expressions observed. Thus, consensus copy-number calls were obtained based on WGS using cnvkit (https://cnvkit.readthedocs.io/en/stable/).

### Analysis of somatic SNVs and spatial chromatin structure

To investigate the correlation between somatic SNVs and spatial chromatin structure in TC, we performed data mining of somatic mutation and Hi-C datasets from cancer genomes. Several previous studies [40–42] have shown that the conformation of mammalian chromatin is conserved across cell types and, to some degree, even across species. Firstly, we identified somatic mutated genes in all three cancer cell types (Additional file 2: Fig. S1A). Statistics about SNV subtypes and genomic distributions are shown in Additional file 2: Fig. S1B–G. Then, we assessed the co-mutational landscape in the three human TC cell lines. Alternatively, for the somatic SNVs, for a specific type of cancer, the 3D contact frequencies were calculated, which evaluated the spatial proximity of two genome segments of these paired co-mutated genes on the basis of the Hi-C datasets. For each given pair of mutated genes that were located on the same chromosome, the linear nucleotide distance was obtained. We did not consider gene pairs that were located on discrepant chromosomes because of the low resolution and sparseness of the interchromosomal Hi-C data. We calculated two types of background for spatial contact frequency: gene-level and overall backgrounds. Additionally, for each co-mutated gene pair, the contact frequencies of the co-mutated genes pairs was also collected via WGS analysis. We obtained three representative empirical distributions by concatenating the frequencies of contact of all the co-mutated gene pairs that occurred in two TC cell lines on the basis of Hi-C and WGS datasets.

### Analysis of gene expression with TAD A/B compartment switches

Besides the analysis of genomic features associated with TADs changes in TC, we also interrogated the correlation between gene expression and A/B switches. The mammalian genome comprises actively transcribed compartment A (also called open state) and inactive compartment B (also called closed state) [43, 44]. Hi-C data from a previous study showed that A/B compartments switching between cancer and normal cells was associated with corresponding alterations in gene expression [45].

### RNA sequencing and data analysis

To isolate poly(A) + mRNA, 10 µg of intact total RNA were prepared using the Dynabeads mRNA Purification Kit (Invitrogen) following the standard protocol of the manufacturer, except that an additional round of purification were performed before the final elution [46–51]. RNA was fragmented and purified, double-stranded cDNA was synthesized, and indexed Illumina libraries were prepared as described for the RNase H libraries, except that 12 cycles of PCR were performed. Each of the poly(A) libraries were sequenced using an Illumina HiSeqX. All libraries were mapped to the human genome (hg19, which includes only chromosomes 1–22, X and Y, and mitochondria) using Tophat (v1.3.3) without gene annotations and with default parameters. Unmated reads were removed; only read pairs with both reads aligning to the genome were retained. After sampling equal numbers of genome-aligning reads, alignment files were generated for RNA-SeQC. HTSeq was used to quantify gene expression (counts) using UCSC knownGene as annotations and used DESeq2 to perform gene differential analysis. We conducted paired-end RNA sequencing, and the number of reads for each of the cell lines was ~ 40 M (~ 6 Gb data).

### Pathway enrichment analysis

Gene Ontology was used to perform enrichment analysis on gene sets. Using the R (version 4.0.1) package *goProfiles* (v3.11), for a specific gene set that contained “*n*” genes, the significance of having “*r*” genes that were involved in a certain function category was computed.

### Statistics

We used the Kolmogorov–Smirnov test to compare the spatial proximities of co-mutated gene pairs in the cancer cells and the backgrounds, and to compare the distances between CNV breakpoints and their closest boundaries of TADs with location-randomized breakpoints. Statistical analyses were performed using R 4.0.2 (https://www.r-project.org/) if not otherwise specified, and two-sided *P* value < 0.05 indicated statistical significance.

The FastQC tool (http://www.bioinformatics.babraham.ac.uk/projects/fastqc/) was used to carry out quality check of the raw data. Source data, additional materials, methods, and resources are detailed in Additional file 1: Table S1.

## Results

### Identification of boundaries of topologically associating domains (TADs) in different types of TC cells

High-throughput chromosome conformation capture (Hi-C) data analysis revealed several boundaries, ranging from 5647 to 5942 and containing different cell types (8305C, BCPAP, and TPC-1, respectively) (Additional file 2: Fig. S2A–F). Moreover, shared signatures of TAD boundaries around our boundary calls were observed across both types of TC cells. A common set of 227 boundaries was identified in both ATC and PTC, with a significant overlap between them (*P* < 0.05). The median distance between the common boundaries was about 541 kb, which is consistent with the median size of TAD usually observed in human cells [22, 52]. Furthermore, to explore whether the chromatin architecture differed between ATC and PTC, these boundaries were intersected with the boundaries of TADs found in cancer cell lines. Thirty-five TADs in ATC and 28 TADs in PTC were observed, exclusively (Additional file 2: Fig. S2G–L).

### Co-mutated gene pairs were spatially close to chromatin conformation in TC

**Fig. 1:**
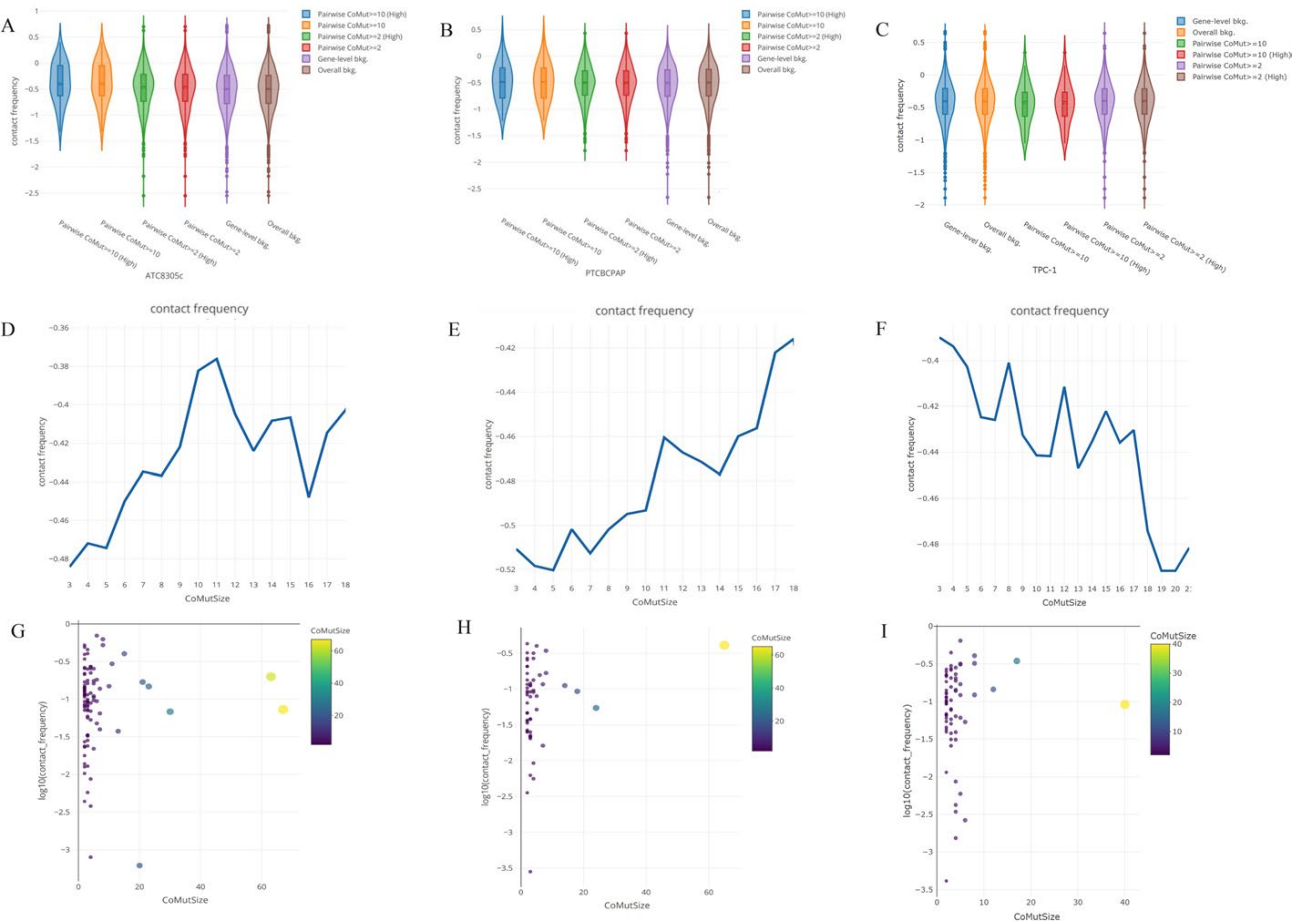
**A**–**C** Spatial proximity of co-mutated gene pairs. Violin plots of the contact frequency distribution of the TAD with various levels of CoMut (high), somatic filtering criteria or on background of gene level or overall level in **A** ATC (8305c), **B** PTC (BCPAP) and **C** PTC (TPC-1) cells, based on Hi-C data. **D**–**I** Spatial proximity of co-mutated gene pairs in TAD. The contact frequency distribution of various CoMut hotspot mutation counts in TAD in (**D**) ATC (8305c), (**E**) PTC (BCPAP), and (**F**) PTC (TPC-1) cells, based on Hi-C data; the contact frequency scatter plot on various CoMut hotspot mutation counts in (**G**) ATC (8305c), (**H**) PTC (BCPAP), and (**I**) PTC (TPC-1) cells based on Hi-C data

Interestingly, the spatial proximities of co-mutated gene pairs in the two types of cancer were significantly greater than in the gene-level and overall backgrounds (Kolmogorov–Smirnov test, *P* = 0.04), while contact frequencies of CoMut (co-mutation gene number within a TAD boundary) > 10 were found to be higher than those of null CoMut (Fig. 1A–C, Additional file 2: Fig. S2A–C). A high-quality CoMut met stringent criteria (QUAL flag = “PASS”) for somatic mutations. To determine whether somatic co-mutated gene pairs occurred in the different TC subtypes, we compared CoMut TADs between ATC and PTC. ATC cells had significantly higher TAD contact frequency around TADs with CoMuts > 10 when compared with PTC cells (both BCPAP and TPC-1 cells). These suggested that somatic co-mutated gene pairs identified in ATC had greater trends of spatial proximities in chromatin structure (*P* < 0.05) (Fig. 1D–I).

**Fig. 2:**
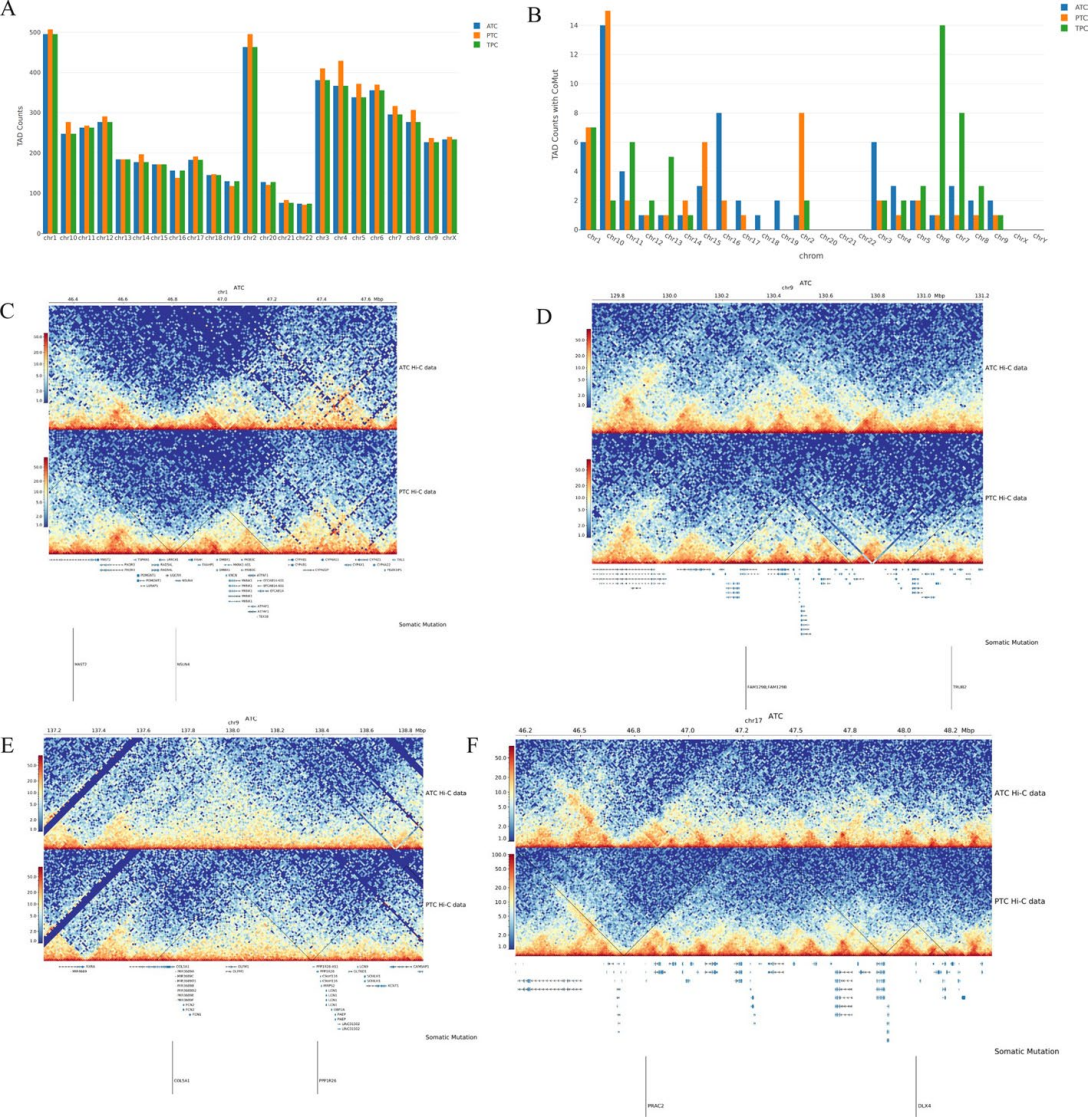
**A**, **B** Spatial proximity of co-mutated gene pairs. Bar plots of the TAD counts **A** without or (**B**) with CoMut hotspots over each chromosome in ATC (8305c), PTC (BCPAP), and PTC (TPC-1) cells. **C**–**F** Spatial proximities of co-mutated gene clusters in TAD based on Hi-C data. CoMut hotspots mutation gene pairs were found in ATC-specific TAD structures

We found spatial co-mutation hotspots in both ATC and PTC (both BCPAP and TPC-1 cells), which are defined to be spatial chromatin loci at which certain genes that are spatially close to each other tend to be co-mutated during tumor initiation and progression. TAD counts with CoMut hotspots over each chromosome are shown in Fig. 2A, B. Furthermore, we investigated ATC and PTC that specifically contained CoMut events within TADs boundaries, by comparing the TAD blocks between different TC subtypes. We found five TAD blocks with CoMut genes/events specific to ATC with certain mutation frequency in The Cancer Genome Atlas Thyroid Cancer (TCGA-THCA) cohort, including CoMut pairs *MAST*/*NSUN4*, *AM129B*/*TRUB2*, *COL5A1*/*PPP1R26*, *PPP1R26*/*GPSM1*/*CCDC183*, and *PRAC2*/*DLX4* (Fig. 2C, F).

### Regulatory elements were enriched near CoMut genes

We examined the regulatory elements surrounding the TADs with CoMut genes that might be involved in cancer initiation and/or progression. Flanking sequences of transcription start sites (TSSs) for CoMut genes within TADs were analyzed using Homer2 to find enriched de novo and known motifs. A total of 37 and 35 motifs were enriched in ATC (8305C cells) and PTC (BCPAP cells), respectively, and 5 motifs were common in both cell types (Additional file 2: Fig. S3A, B). We found that, for the majority of ATC and PTC cells, the HOXA10 and HIF2α signals near the TSSs of CoMut genes within TADs were significantly stronger than those at the background. Additionally, we found enriched ATC-specific motifs, such as SMAD4 and GLI2 (Additional file 2: Fig. S3C, D).

### BRAF V600E and TERT C228T mutations

We used GTAK Mutect2 to check the *BRAF* mutation, and tools of GTAK Haplotype Caller and Varscan2 to analyze the *TERT* promoter mutation; we also used samtools and BedTools to analyze the associations of the coverage of both with Hi-C TAD domain. *BRAF V600E* was found in both 8305C and BCPAP cells. Although BCPAP cells had 99.81% mapped reads, the *TERT C228T* variant was not covered by the WGS analysis of BCPAP cells as expected. It is a limitation of the next-generation sequencing (NGS) platform in sequencing this region. Then, we checked both *BRAF V600E* and *TERT C228T* mutations in Hi-C results to search related spatial chromatin structure, and found that the locations of *BRAF V600E* and *TERT C228T* mutations both fell into the TAD domains (at 10 kb resolution) in both ATC (TAD chr5: 910000–1540000 overlapped with *TERT* promoter mutation, and TAD chr7: 140360000–141350000 overlapped with *BRAF V600E*) and PTC cells (TAD chr5: 1170000–1350000 overlapped with *TERT* promoter mutation, and TAD chr7: 140370000–140710000 overlapped with *BRAF V600E*).

### Copy number variations (CNVs) in different TC subtypes

**Fig. 3:**
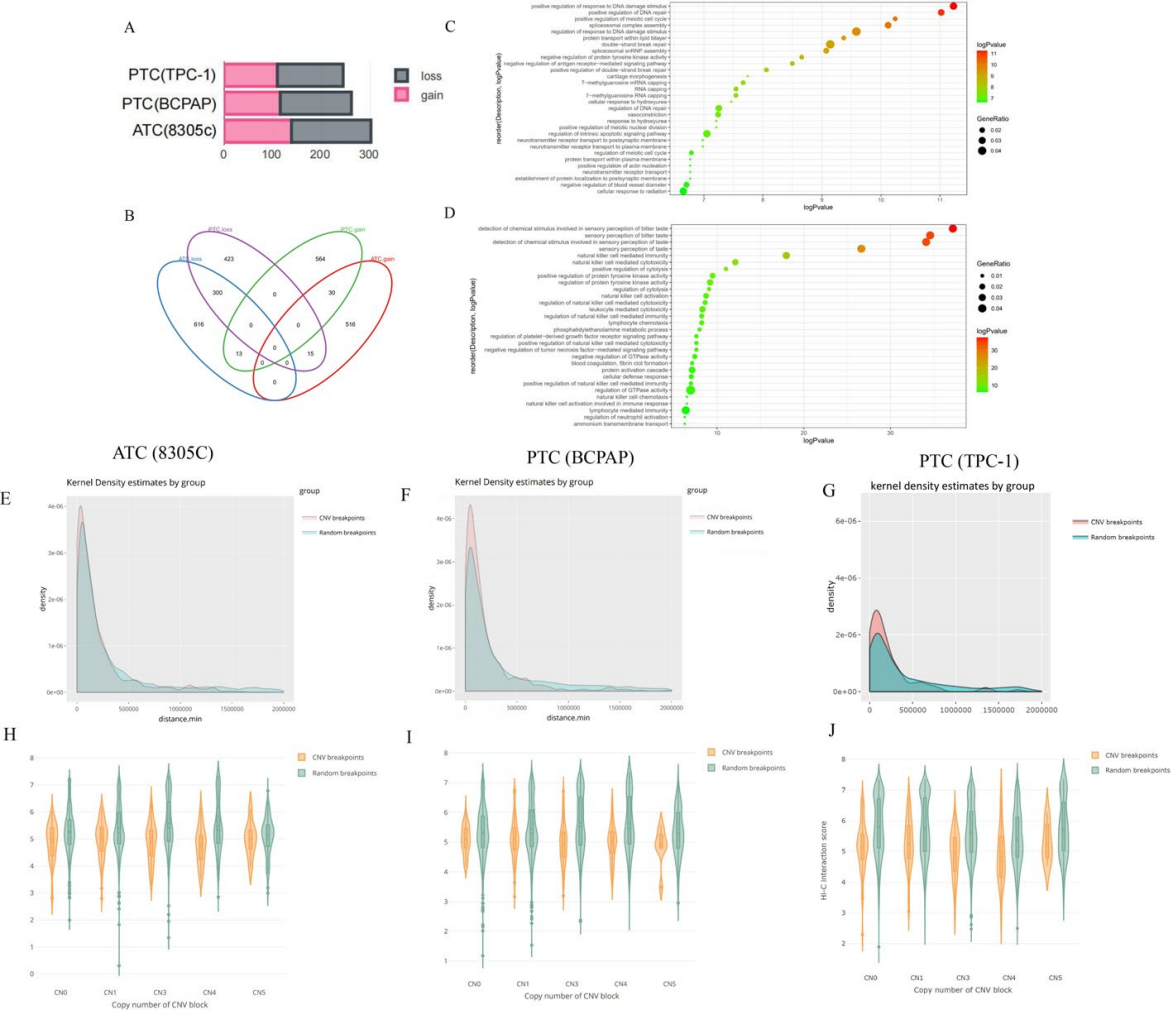
**A**–**D** ATC-specific copy number variations and annotated biological functions. **A** CNV gain and loss events in ATC (8305C) and both PTC (BCPAP and TPC-1) cell lines; **B** intersection plot of multiple subgroups of CNV statuses in ATC and both PTC cells; **C**, **D** significantly enriched Gene Ontology (biological functions) in genes associated with ATC-specific CNVs gain and loss events. **E**–**J** CNV breakpoints associated with TAD boundaries. The distribution plots of the distance between all CNV breakpoints (red line) and random sites (green line) to their nearest TAD boundaries in **E** ATC (8305C), and **F** PTC (BCPAP), and **G** PTC (TPC-1) cells; distributions of distances (kb) from various CNV events to the nearest TAD boundaries in **H** ATC (8305C), **I** PTC (BCPAP), and **J** PTC (TPC-1) cells

We found that ATC (8305C) cells had more CNV changes (301 events in total, including 138 gains and 163 losses) compared with PTC (BCPAP) (261 events in total, including 115 gains and 146 losses) and PTC (TPC-1) cells (244 events in total, including 109 gains and 135 losses) (Fig. 3A). Overlapped CNVs in ATC (8305C) and PTC (BCPAP) cells involved 300 genes of loss events and 30 genes of gain events (Fig. 3B). Overlapped CNVs in ATC (8305C) and PTC (TPC-1) cells involved four genes of loss events and two genes of gain events. Both PTC cells also had fewer common CNVs (two shared losses and two shared gains). Then, we performed GO and KEGG/Reactome enrichment analysis for subtype-shared or subtype-specific CNV-involved genes. The functions of ATC-specific genes mostly involved in CNV gain included positive regulation of response to DNA repair, DNA damage stimulus, meiotic cell cycle, and spliceosome complex assembly and negative regulation of protein tyrosine kinase activity, etc. (Fig. 3C). Positive regulation of protein tyrosine kinase activity, regulation of cytolysis, leukocyte-mediated cytotoxicity, and natural killer cell-mediated immunity and negative regulation of GTPase activity were related to genes involved in CNV loss events (Fig. 3D).

### Spatial genome disorganization was associated with CNV

An integrated analysis that combined Hi-C and whole-genome sequencing (WGS) data in ATC and PTC cells was applied. It was found that CNV breakpoints significantly overlapped with boundaries of TADs in all cancer subtypes (Fig. 3E–G). Together, we identified 5647 boundaries of TADs at 50-kb resolution, and 301 CNV breakpoints in ATC cells. Most often, CNV breakpoints took place close to boundaries of TADs, with a total of 158 (52.5%) CNV breakpoints located within 120 kb of TAD boundaries. The distances between CNV breakpoints and their closest TAD boundaries were significantly shorter than location-randomized breakpoints (Kolmogorov–Smirnov test, *P* < 2.2 × 10^−16^; Fig. 3E). The distance of CNV breakpoints for each CN status (0, 1, 3, 4, or 5) between TAD boundaries was shorter than that of location-randomized breakpoints (Fig. 3H). The results were similar in both PTC cell lines (Fig. 3I, J), confirming the correlation between CNV breakpoints and boundaries of TADs in TC cells.

### Boundaries of TADs were influenced by discrepant types of somatic structural variation (SV) in cancer genomes

**Fig. 4:**
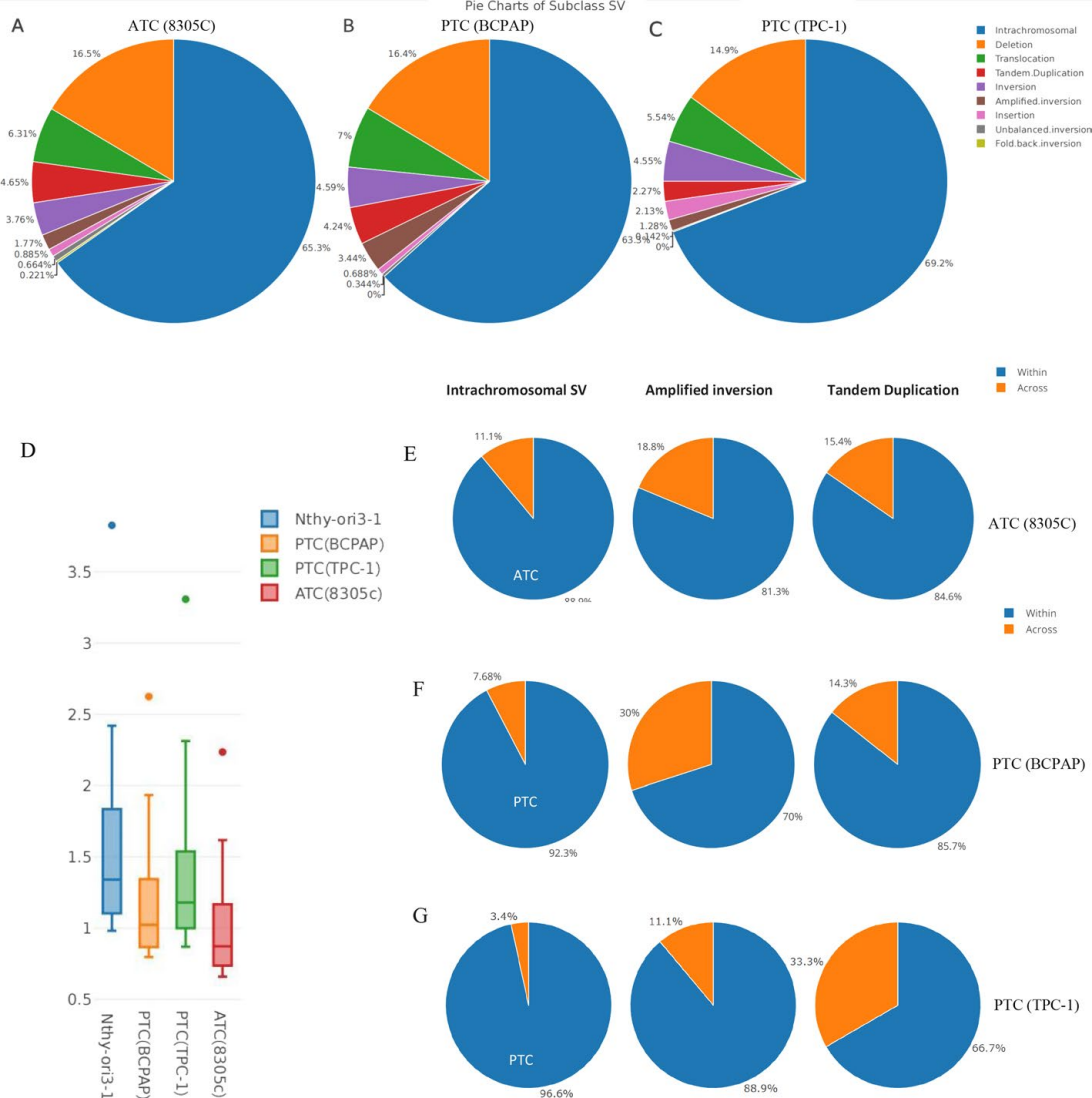
**A**–**C **SV subtypes identified on the basis of WGS. The distribution of SV subtypes in **A** ATC (8305C), **B** PTC (BCPAP), and **C** PTC (TPC-1) cells. SVs were identified by DELLY2. **D** Differences in TAD changes between ATC (8305C), PTC (BCPAP and TPC-1), and normal thyroid cell lines. The ratio of short-range to long-range TAD blocks in ATC, PTC, and normal cell-lines. **E**–**G** SV breakpoints associated with TAD boundaries. The distribution of SV subtypes across (span-SV) or within nearest TAD boundaries (within-SV) in **E** ATC (8305C), **F** PTC (BCPAP), and **G** PTC (TPC-1) cells

To understand the effects of SVs on boundaries of TADs in human cancers, we used 904 and 872 high-confidence somatic SVs breakpoints identified by Delly2 in ATC (8305C) and both PTC (BCPAP and TPC-1) cell lines and then classified SVs into nine classes, including inversions, duplications, deletions, or complex rearrangements as described previously [53] (Fig. 4A–C). Subtypes of SV in ATC and PTC were comparable in numbers. Furthermore, we also categorized SVs into subgroups according to length of SV range: SVs with genome length > 2 Mb and < 2 Mb (long-range and short-range SVs, respectively). The ratio of short-range to long-range TAD block in ATC (8305C), PTC (BCPAP and TPC-1), and normal cell-lines is shown in Fig. 4D. We found that ATC had more often significantly shorter TADs than PTC. The majority of inversions, deletions, and duplications were categorized as short-range SVs; however, complex events tended to be of longer range. We focused on short-range SVs because long-range SVs could influence multiple boundaries due to the genome length of the events. SVs that influenced the boundaries of TADs were identified as ones that spanned the whole length of a boundary (around 75 kb) or within the whole TADs. The percentage of SVs with length ≤ 2 Mb within TADs (solid) and across TADs (shaded) for discrepant types of SVs was analyzed. We referred to SVs that occurred across TADs as span-SVs, while SVs that occurred within TADs were referred to as within-SVs. Seventy span-SV and 537 within-SV events were identified in ATC, and 49 span-SV and 510 within-SV events were identified in PTC (Fig. 4E–G). Only intrachromosomal SVs, amplified inversions, and tandem duplications spanning or within TADs were found in ATC and PTC (Fig. 4E–G). In contrast, we observed that span-SV events of amplified inversions occurred more frequently in PTC than in ATC (*P* < 0.05), whereas within-SV events of intrachromosomal SVs occurred more often in ATC than in PTC (*P* < 0.05).

### Profiles of regulatory elements and heterochromatic states around TAD boundaries

**Fig. 5:**
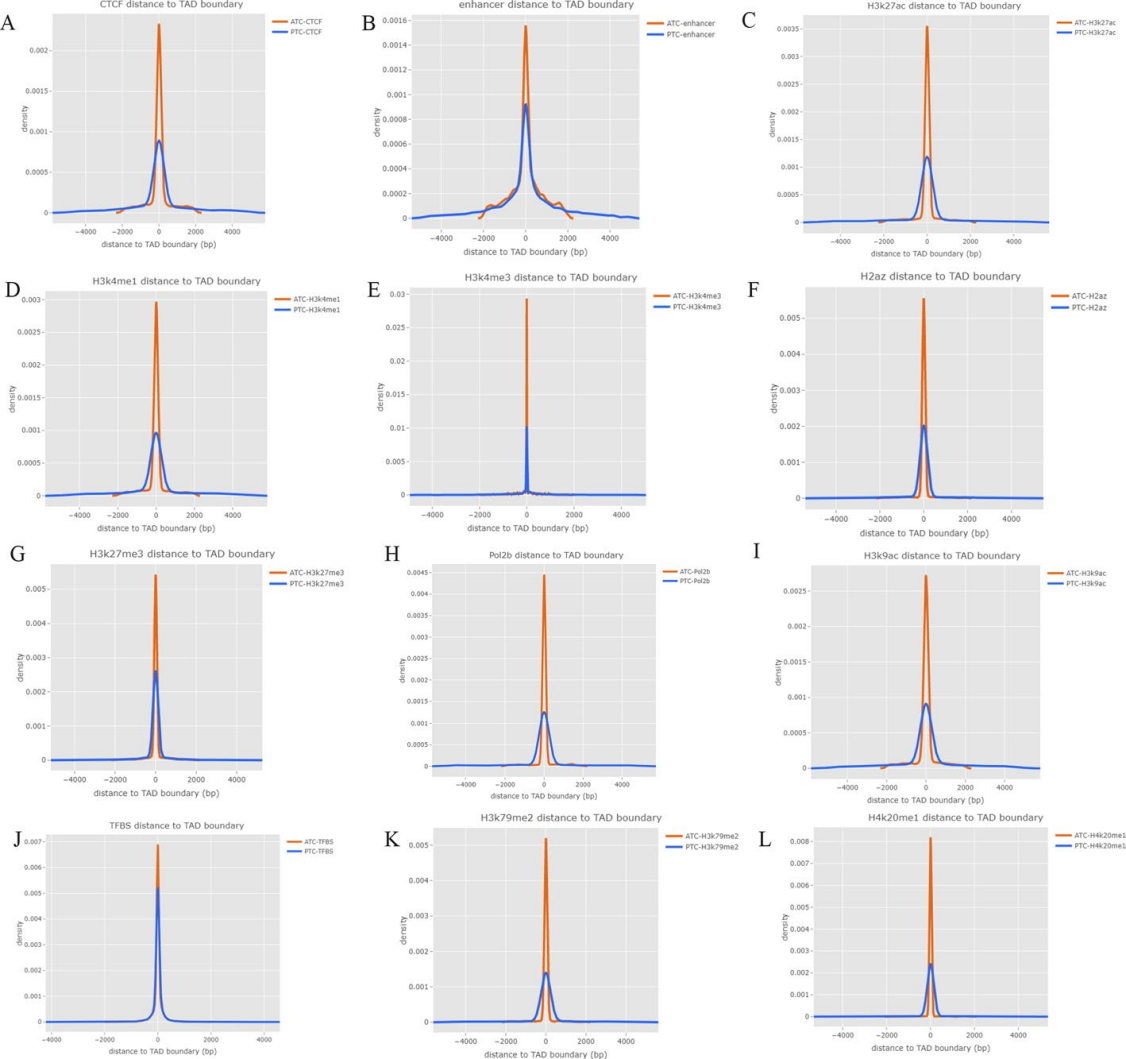
**A**–**F** Density of distance between gene regulatory features and heterochromatic states around TAD boundaries. To explore epigenetic mechanisms, enrichment of regulatory elements and motifs from various cell types that have previously been profiled by the Encyclopedia of DNA Elements (ENCODE) consortium and the Roadmap Epigenome project, which are involved in DNA methylation, chromatin remodeling, regulation by noncoding RNAs, and binding of regulatory proteins, and that include **A** 11-zinc finger protein (CTCF), **B** enhancers, **C** H3K27ac, **D** H3K4me1, **E** H3K4me3, and **F** H2az, was analyzed around the TAD boundaries. **G**–**L **Density of distance between gene regulatory features and TAD boundary. To explore epigenetic mechanisms, enrichment of regulatory elements and motifs from various cell types, which had previously been profiled by the Encyclopedia of DNA Elements (ENCODE) consortium and the Roadmap Epigenome project and which included **G** H3K27me3, **H** Pol2b, **I** H3K9ac, **J** TFBS, **K** H3K79me2, and **L** H4K20me1, was analyzed around the TAD boundaries

**Fig. 6:**
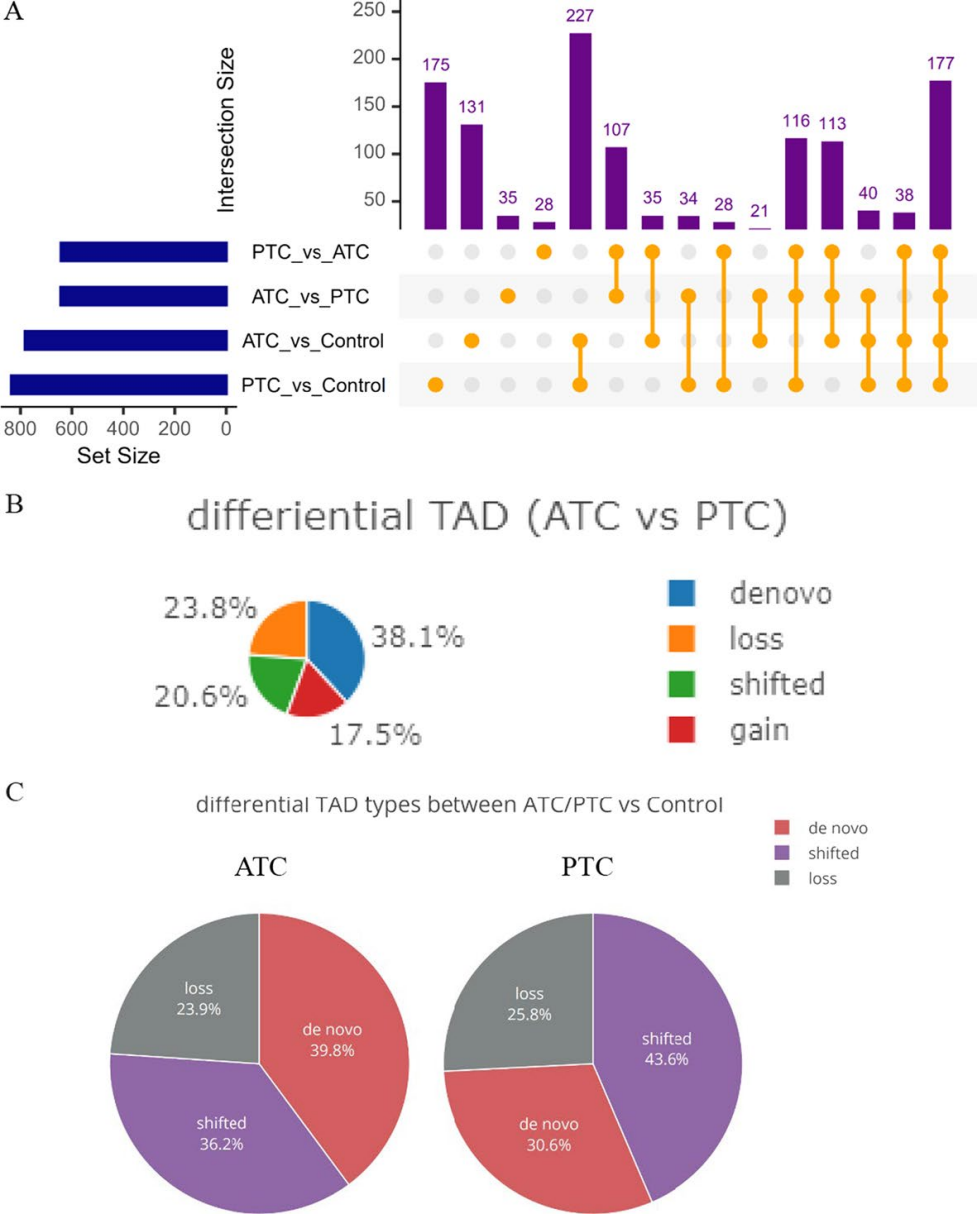
**A**–**C** Differences in TAD changes between ATC, PTC, and normal thyroid cell lines. **A** Comparison of differential TAD blocks among ATC, PTC, and normal cell lines; **B** TAD changes (de novo, loss, shifted, and gain) between ATC and PTC cell lines; **C** Global TAD changes (de novo, shifted, and loss) in ATC and PTC versus normal

**Fig. 7:**
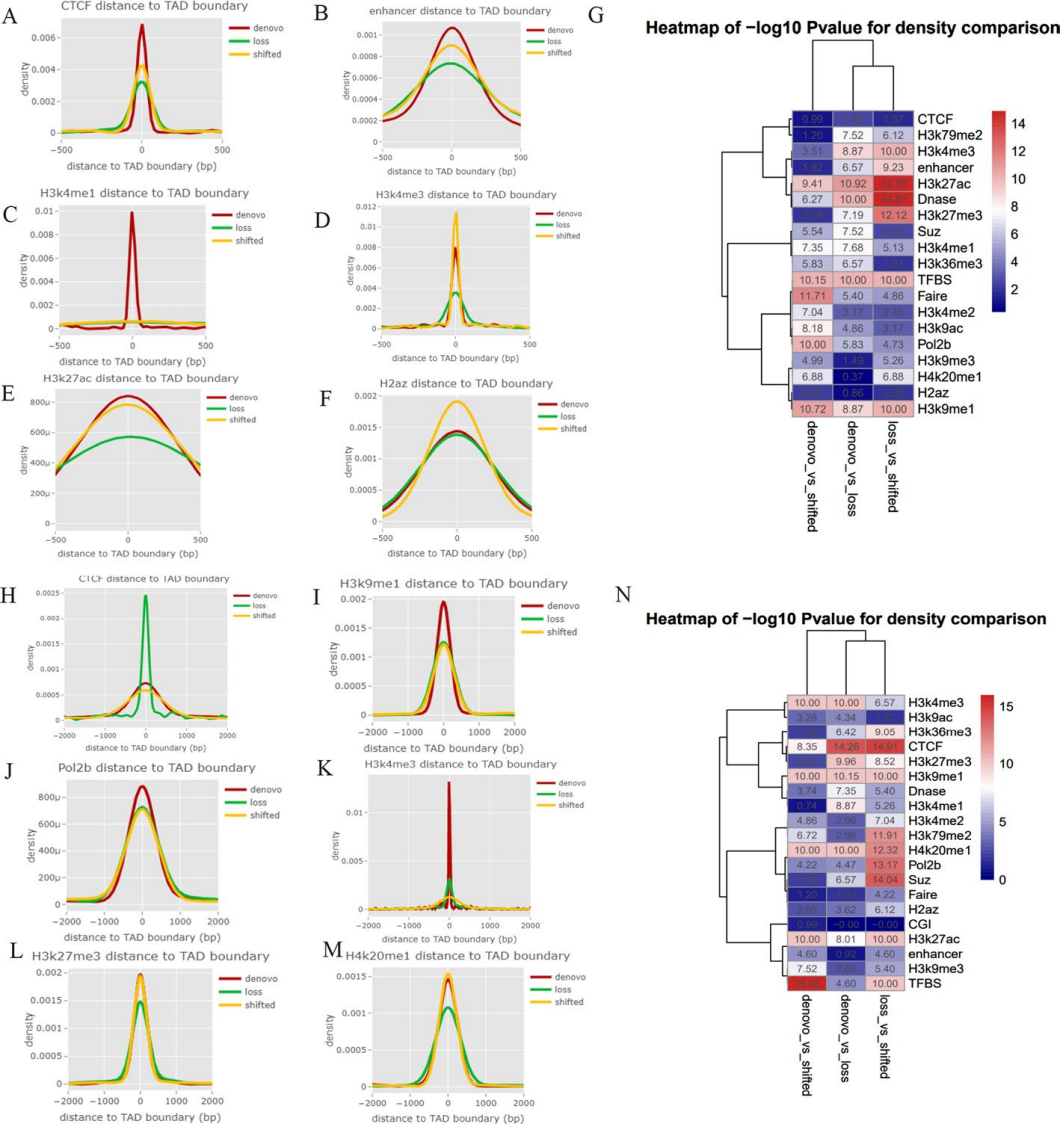
**A**–**G** Density of distance between regulatory features and changed TADs in ATC cells. Various factors and regulatory and epigenetic markers around changed TADs in ATC compared with normal cells are shown in **A**–**F**, in which different TAD change modes, including de novo, loss, and shifted, were compared; **G** The significance of differences between two TAD change modes (de novo versus shifted, de novo versus loss, and loss versus shifted) for epigenetic markers are visualized using heat maps. **H**–**N** Density of distance between regulatory features and changed TAD in PTC cells. Various factors and regulatory and epigenetic markers around changed TADs in PTC compared with normal cell line are shown in **H**–**M**, in which different TAD change modes, including de novo, loss, and shifted are compared. **N** The significance of differences between two TAD change modes (de novo versus shifted, de novo versus loss, and loss versus shifted) for epigenetic markers are visualized using heat maps

To investigate the interaction between chromatin conformation and epigenetic profiles, we investigated the enrichment of enhancers, promoters, CpG islands, DNase I-hypersensitive, and CCCTC-binding factor (CTCF)-binding sites, as well as heterochromatic regions around boundaries and active transcription initiation sites from several cell types that the Roadmap Epigenome project and the Encyclopedia of DNA Elements (ENCODE) consortium have previously profiled. We found that active promoter marks, CTCF-binding sites, and heterochromatin states were mostly enriched at the boundaries in both ATC and PTC, and that those markers in ATC had significantly stronger signals than in PTC (*P* < 10^–15^) (Fig. 5). These findings indicated that ATC had more changes in dynamic regulations that interplayed with TADs. To understand how TAD changes were associated with these regulatory signatures, we compared identified TADs between ATC and PTC with normal cells as control, and defined cell-specific TAD boundaries and four TAD changes modes, including de novo, loss, shifted, and gain. Finally, we identified 35 ATC-specific TADs and 28 PTC-specific TADs (Fig. 6A). By comparing TAD boundaries of ATC with those of PTC, 38.3% de novo changes, 23.8% loss changes, 20.6% shifted TADs, and 17.5% gained TADs were observed in ATC (Fig. 6B, C). The baselines of TAD changes in ATC and PTC compared with those in normal cells are shown in Fig. 6C. We also compared the epigenetic markers and regulatory signatures around TAD boundaries in each TAD change mode (Fig. 7A–N). We found that CTCF, enhancers, and H3K4me1 signals were significantly enriched in de novo TAD changes in ATC cells (Fig. 7A), while loss events or shifted TADs had the weakest signals. In contrast, PTC-specific TADs changes presented stronger signals in CTCF than in loss TAD changes (Fig. 7H). The significant differences between pairs of two TAD change modes (de novo versus shifted, de novo versus loss, and loss versus shifted) for epigenetic markers were visualized as heat maps and density plots surrounding the TAD boundaries (Fig. 7A–N).

### Gene expression associated with TAD A/B compartment switches

**Fig. 8:**
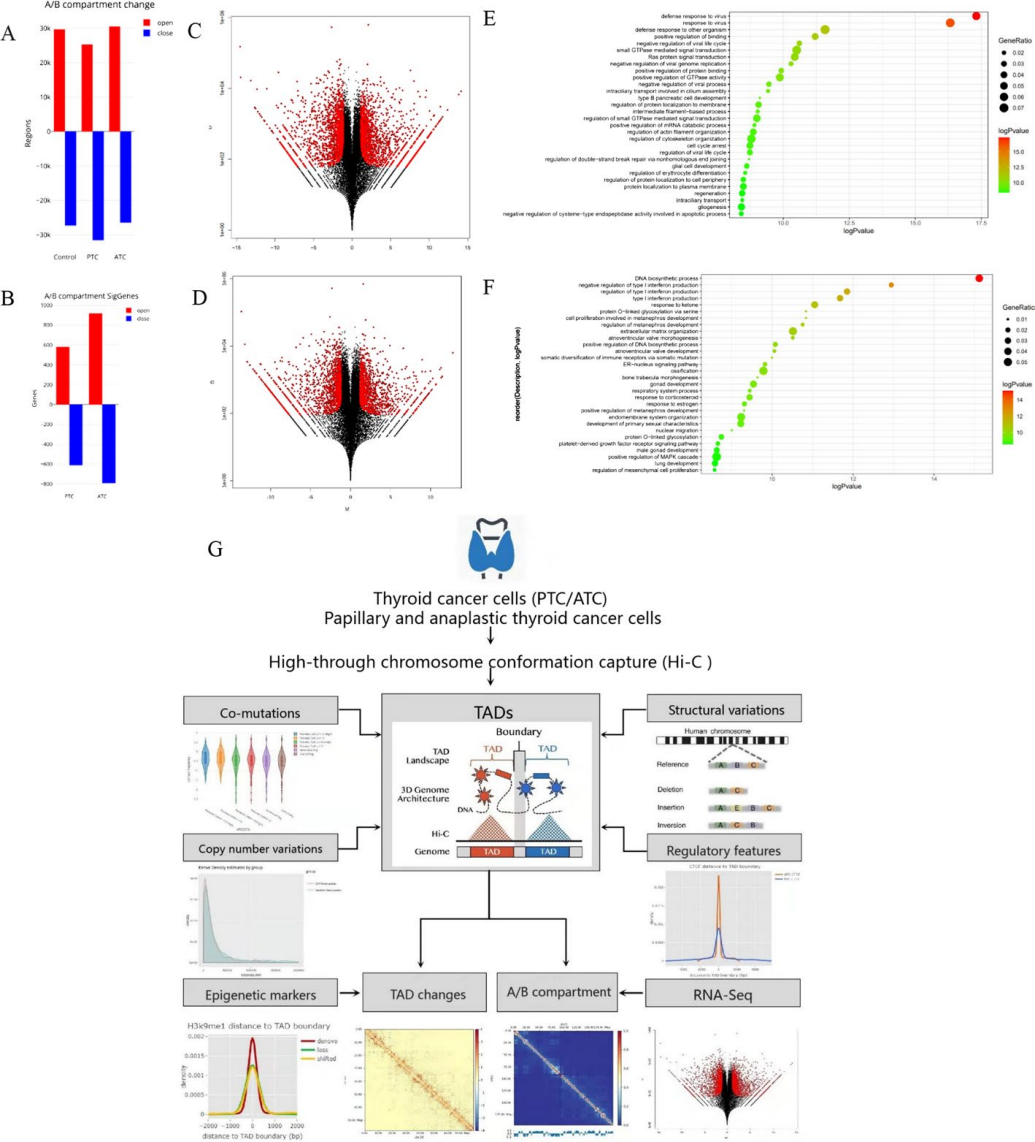
Gene expressions associated with TAD A/B compartment switches. **A** A total of 30,539, 25,335, and 29,647 A compartments (open state) were identified in ATC, PTC, and normal cells, respectively; a total of 26,420, 31,614, and 27,314 B compartments (closed state) were identified in ATC, PTC, and normal cells, respectively. **B** By comparing ATC with normal cells, a total of 0.6% genomic regions switched from compartment A (open state) in normal cells to compartment B in ATC and were associated with downregulated gene expression. In contrast, 0.9% of genomic regions exhibited the opposite switching from compartment B in normal cells to compartment A in ATC and were associated with upregulated gene expression. **C**, **D** Compared with normal cells, a total of 3562 genes (1727 downregulated and 1835 upregulated genes) and 2786 genes (1308 downregulated and 1478 upregulated genes) were differentially expressed in ATC and PTC, respectively. **E**–**F** GO and pathway enrichment analysis for genes switched **E** from compartment A to B, or **F** from compartment B to A. **G** Overall study scheme

By exploiting the structure of long-range correlations between open and closed compartments, we tried to explore the variations in gene expression. A total of 30,539, 25,335, and 29,647 A compartments (open state) were identified in ATC, PTC, and normal cells, respectively; a total of 26,420, 31,614, and 27,314 B compartments (closed state) were identified in ATC, PTC, and normal cells, respectively (Fig. 8A and Additional file 2: Fig. S4). Comparing ATC with normal cells, it was found that 0.6% of genome regions switched from compartment A in normal cells to compartment B in ATC and were correlated with downregulated expression of genes (Fig. 8B). In contrast, 0.9% of genome regions showed the opposite switching from compartment B in normal cells to compartment A in ATC and were correlated with upregulated expression of genes. Accompanied with A/B compartment analysis, a total of 3562 genes (1727 downregulated and 1835 upregulated genes) and 2786 genes (1308 downregulated and 1478 upregulated genes) were differentially expressed in ATC and PTC, respectively (Fig. 8C, D), compared with normal cells using RNA sequencing. Combining RNA-seq gene expression data with findings of A/B compartment switches analysis, a total of 0.9% genome regions switched from compartment A in normal cells to compartment B in PTC, resulting in downregulation of genes. In contrast, 0.5% of genome regions showed the opposite switching, from compartment B in normal cells to compartment A in PTC cells, and were correlated with upregulation of genes.

We further performed a differential expression analysis on RNA-seq data to explore gene expressions in ATC, PTC, and normal cells, respectively. Combining RNA-seq data with A/B compartment switch between normal and ATC cells, gene expression exhibited the same propensity of switch from compartment A to B; 267 (15.5%) of total downregulated genes showed A-to-B compartment switch and 360 (19.6%) of total upregulated genes showed B-to-A compartment switch. GO and pathway enrichment analysis showed that genes with switch from compartment A to B were mostly associated with defense response to virus, *Ras* protein signal transduction, cell–cell junction, and cell cycle arrest, while genes with switch from compartment B to A were associated with negative regulation of type I interferon production, DNA biosynthetic process, cell proliferation, which was involved in metanephros development, and positive regulation of DNA biosynthetic process (Fig. 8E, F).

## Discussion

Higher-order structure of chromatin is often disorganized in cancers. While several genetic and epigenetic discrepancies have been identified between normal and TC tissues, alterations in higher-order chromatin organization during TC genesis and progression have not been fully characterized. Herein, we integrated WGS and Hi-C data to reveal the dynamic higher-order chromatin organization in TC. We comprehensively explored the interactions between chromatin conformations involved in the genetic regulation of SNVs, CNVs, and SVs, and interpreted the patterns of regulatory elements and epigenetic markers around the TADs boundaries. We found that chromatin interactions, genetic alterations, and gene regulation were associated with cancer heterogeneity and subtypes in TC, and indicated the enrichment of CoMut with Gli2, HOXA10, and HIF2α around TADs in TC.

For the first time, we used 3D genome sequencing technology to comprehensively clarify the rules of chromosomal spatial regulation in two TC subtypes, integrating the perspectives of multiple omics analyses (Fig. 8G). We presented a framework that integrated Hi-C, WGS, and transcriptome sequencing to systematically detect the associations between the somatic co-mutations of cancer-related genes, CNVs, SVs, and high-order conformation of chromatin in two TC subtypes. We also observed differential patterns of genomic aberrations surrounding the TADs, such as CNV gains/losses and regulatory element motifs associated with tumorigenesis and immune evasion propensity, and identified more newly established three-dimensional chromatin structural domains in poorly differentiated TC. We accurately captured the landscape of genomic aberrations of TC cells, which were largely different from the normal thyroid cells. Our results may contribute to comprehensive understanding of the characteristics of genes associated with the prognosis and recurrence risk of TC.

We found five TAD blocks with CoMut genes/events specific to ATC with certain mutation frequency, including CoMut pairs *MAST*/*NSUN4*, *AM129B*/*TRUB2*, *COL5A1*/*PPP1R26*, *PPP1R26*/*GPSM1*/*CCDC183*, and *PRAC2*/*DLX4*. Microtubule-associated serine-threonine (*MAST*) kinase is associated with gene rearrangement in breast cancer and related pre-invasive lesions [54], while Gene Set Enrichment Analysis (GSEA) indicated that *NSUN4* was associated with methylation and demethylation processes in hepatocellular carcinoma [55]. *FAM129B* is a new regulator of Wnt/β-catenin-dependent phenotypes in melanoma cells [56], and *TRUB2* is an age-associated cancer marker [57]. We observed that *HOXA10* and *HIF2* genes were commonly found surrounding the TADs with CoMut genes. Differential expression of *HOXA* genes has been previously reported in TC [58, 59]. Furthermore, a study [60] reported that the overexpression of HIF-1/2α was correlated with the genesis of PTC, thus serving as potential biomarkers for PTC. These observations led us to hypothesize that such regulators might be key factors that contributed to gene co-mutations via mediations of high-order chromatin conformation, for instance, via forming long-range loops of chromatin or insulating epigenetic signals.

Since cancer is frequently correlated with genome alterations such as aneuploidy, it is vital to analyze CNV to depict the disorganization of the cancer genome and determine its functional consequences. Our results revealed CNV losses in genes involved in function mediated by natural killer cells, which are a key antitumor effector to overcome the immune-evasion mechanisms used by cancers and to achieve complete tumor eradication in a manner coordinated and synergistic with other antitumor cells [61]. Our findings suggested that ATC might have a greater propensity for immune evasion leading to poor prognosis than PTC. A recent study suggested that CNVs might rebuild new TAD boundaries in cancer genome [62]; we therefore further explored the relationship between TADs and CNVs in TC, to depict the spatial disorganization of the cancer genome affected by CNV and interpret how CNV impacted TC via higher-order chromatin interactions. Our findings supported the assumption that CNV breakpoints were prone to take place near boundaries of TADs in cancer cells and that CNV might induce the formation of new TADs with boundaries near breakpoints of CNVs.

Complex rearrangements such as chromothripsis and other changes that cover SV break-ends with concomitant inversions, deletions, or duplications are responsible for disruption of chromatin folding domains [63]. Our findings suggested that duplication, inversion, and complex intrachromosomal SVs tended to take place within the same TAD, while amplified inversion tended to span more regions across discrepant TADs in PTC than in ATC.

Epigenetic changes are characteristics that are heritable and that affect the phenotype by interplaying with the expression of genes without influencing the sequence of DNA; they include remodeling of chromatin, methylation of DNA, regulation by noncoding RNAs, and binding of transcription factors and regulatory proteins [e.g., 11-zinc finger protein (CTCF) and brother of the regulator of the imprinted site (BORIS)] [64, 65]. Many of these mechanisms also influence the states of chromatin and may be responsible for the variations in cancer. We revealed that the genome features of boundaries of TADs across different human TC subtypes might dynamically regulate the specific gene activations or inhibitions related to poor prognosis in patients with ATC. These modifications are linked to open or closed chromatin conformation, and they influence gene access to regulatory proteins and transcription factors. As a result, cells may use histone alterations to dynamically regulate their gene expression [66].

Regulatory elements, such as CTCF, enhancers, and H3K4me1 signals were significantly enriched in de novo TAD changes in poorly differentiated TC, while loss events or shifted TADs had the weakest signals. These phenomena showed that the genomic characteristics of boundaries of TADs across discrepant human TC subtypes might dynamically regulate the specific gene activations or inhibitions related to poor prognosis in patients with ATC. These modifications correlated with closed or open conformations of chromatin and drove differential access of genes to regulatory proteins and transcription factors. Thus, cells can dynamically use histone alterations to regulate their gene expression. Furthermore, the switches between the A and B compartments were correlated with specific subtypes of gene expression profiles in ATC compared with PTC. A/B compartment switches between TC and normal cells exhibited specific gene expression patterns, which were in accordance with RNA-seq data. Inactivation of genes related to cell cycle arrest pathway may impact tumor cell proliferation. Reversely, genes related to cell proliferation and DNA replication were activated. These dysfunctional pathways involved with inactive/active genes might lead to the malignant transformation of ATC mediated by chromatin conformation.

We tried to capture the landscape of genomic aberrations, including CNVs and SVs, in both TC cells compared with normal thyroid cells, to comprehensively understand the characteristics of genes correlated with the prognosis and recurrence risk of TC. By combining Hi-C with genome and RNA sequencing, we uncovered the relationships between TADs, SNVs, complex SVs, and CNVs in TC. We observed differential patterns of genomic aberrations surrounding the TADs and identified more newly established three-dimensional chromatin structural domains in poorly differentiated TC.

In this study using data newly generated by ourselves, we comprehensively analyzed four representative cell lines derived from human PTC (BCPAP and TPC-1 cells), ATC (8305C cells), and normal thyroid tissues to reveal the spatial gene alterations and regulations in TC. Notably, the PTC cell line BCPAP is derived from a poorly differentiated PTC (PDPTC) [15]. To further improve the representativeness of our findings, we have analyzed another PTC cell line, TPC-1, with comparisons with the PDPTC and ATC cell lines. Other TC cell lines need to be analyzed to further validate the findings. Moreover, it is unclear whether the findings based on stabilized cell lines are generalizable to patients. It would be important to perform further studies on tumor tissues from patients in the future. This study with newly established and validated methodology in TC research lays an important foundation for future studies of patients’ tumor tissues.

While the use of cell lines was a limitation of our study, our findings provide important evidence for and highlight the need for further relevant investigations on mechanism of action and validation in human cancer tissues. The contributions of our findings to precision cancer medicine regarding individualized prediction of survival and treatment benefits also warrant further exploration.

## Conclusions

We presented a comprehensive analysis of the genomic and transcriptomic alterations associated with cancer heterogeneity and subtypes in TC (Fig. 8G). Our findings imply that chromatin interactions and gene alterations and regulations are largely heterogeneous in TC. Gene expression was orchestrated by complex epigenetics and regulatory elements. CNVs and complex SVs may function in the TC genome by interplaying with TADs, and are largely different between ATC and PTC. Complexity of TC genomes, which are highly organized by 3D genome-wide interactions mediating mutational and structural variations and gene activation, may have been largely underappreciated. Our comprehensive analysis may provide key evidence and targets for more customized diagnosis and treatment of TC.

## Supplementary Information

**Additional file 1: Table S1.** Key resources

**Additional file 2: Figure S1.** Summary of somatic mutations and distribution of somatic mutations in ATC and PTC cell lines. **Figure S2.** Hi-C interaction heat-maps in ATC and PTC cell lines. **Figure S3.** Motifs analysis for TAD regions with CoMut. **Figure S4.** TAD A/B compartment switches in ATC cells ordered in each chromosome.

## Data Availability

All data and materials are available in links provided in Additional file 1: Table S1.

## Abbreviations

ATC: Anaplastic thyroid cancer
PTC: Papillary thyroid cancer
TC: Thyroid cancer
TADs: Topologically associating domains
CoMuts: Co-mutations
Hi-C: High-throughput chromosome conformation capture
CNVs: Copy number variations
SVs: Structural variations
WGS: Whole-genome sequencing
