# Supplementary material for: Three-dimensional genome landscape comprehensively reveals patterns of spatial gene regulation in papillary and anaplastic thyroid cancers: a study using representative cell lines for each cancer type
Source: Cell Mol Biol Lett. 2023 Jan 6;28:1. doi: 10.1186/s11658-022-00409-6 (PMC9825046; doi:10.1186/s11658-022-00409-6)
Supplement: Supplementary file 1 — Additional file 1: Table S1. Key resources [file 11658_2022_409_MOESM1_ESM.docx]

**Additional file 1: Table S1.** Key resources

| **Deposited Data** |  |  |
| --- | --- | --- |
| **Hi-C Sequencing Data** |  |  |
| ATC Hi-C data |  | <http://58.33.13.154:8001/release.results/>ZLL/HiC/ATC |
| PTC Hi-C data |  | <http://58.33.13.154:8001/release.results/>ZLL/HiC/PTC |
| Nthy-ori 3-1 Hi-C data |  | <http://58.33.13.154:8001/release.results/>ZLL/HiC/NC |
| **Transcriptome sequencing** |  |  |
| ATC mRNA-Seq |  | <http://58.33.13.154:8001/release.results/>ZLL/RNA/ATC |
| PTC mRNA-Seq |  | <http://58.33.13.154:8001/release.results/>ZLL/RNA/PTC |
| Nthy-ori 3-1 mRNA-Seq |  | <http://58.33.13.154:8001/release.results/>ZLL/RNA/NC |
| **Whole-genome sequencing** |  |  |
| ATC WGS data |  | http://58.33.13.154:8001/release.results/ZLL/WGS/ATC |
| PTC WGS data |  | <http://58.33.13.154:8001/release.results/>ZLL/WGS/PTC |
| Nthy-ori 3-1 WGS data |  | <http://58.33.13.154:8001/release.results/>ZLL/WGS/NC |
| **Experimental Models: Human thyroid cancer and normal thyroid cell lines** | | |
| PTC (BCPAP) | DSMZ database | <https://www.dsmz.de/collection/catalogue/details/culture/ACC-273> |
| PTC (TPC-1) | Expasy database | https://web.expasy.org/cellosaurus/CVCL_6298 |
| ATC (8305c) | ATCC database | https://www.atcc.org/products/crl-3351 |
| Nthy-ori 3-1 | Expasy database | https://web.expasy.org/cellosaurus/CVCL_2659 |
| **Software and Algorithms** |  |  |
| Hi-C analysis pipeline | HiCExplorer (v40) | <https://github.com/deeptools/HiCExplorer> |
| Whole-genome sequencing analysis | GATK4 (v4.1.8.1) | <https://github.com/broadinstitute/gatk> |
| Hi-C visualization tool | pygenometracks (3.0) | https://github.com/deeptools/pyGenomeTracks |
| Bam processing | SAMtools (v1.10) | <https://github.com/samtools/samtools> |
| Duplicate filtering | Picard (v2.23.4) | <https://github.com/broadinstitute/picard> |
| Somatic mutations | Mutect2 | <https://software.broadinstitute.org/gatk> |
| Copy-number calls | cnvkit (v0.9.7) | <https://github.com/etal/cnvkit> |
| Structural variation | DELLY (v0.8.3) | <https://github.com/dellytools/delly> |
| Structural variation | Lumpy (v0.3.4) | <https://github.com/arq5x/lumpy-sv> |
| RNA-Seq quality control | RNA-SeQC | [http://www.broadinstitute.org/cancer/cga/tools/rnaseqc](https://link.jianshu.com/?t=http://www.broadinstitute.org/cancer/cga/tools/rnaseqc/RNA-SeQC_v1.1.8.jar) |
| RNA-Seq Genome alignment | Tophat (v1.3.3) | <http://ccb.jhu.edu/software/tophat/index.shtml> |
| Gene expression | HTSeq (v[0.12.4](https://htseq.readthedocs.io/en/master/history.html#version-0-12-4)) | <http://htseq.readthedocs.io/> |
| Gene differential analysis | DESeq2 (v1.28.1) | <http://www.bioconductor.org/packages/release/bioc/html/DESeq2.html> |
| Gene enrichment analysis | goProfiles (v1.50.1) | <http://www.bioconductor.org/packages/release/bioc/html/goProfiles.html> |
| Statistics | R (version 4.0.1) | <https://www.r-project.org/> |
