# Supplementary material for: Three-dimensional genome landscape comprehensively reveals patterns of spatial gene regulation in papillary and anaplastic thyroid cancers: a study using representative cell lines for each cancer type
Source: Cell Mol Biol Lett. 2023 Jan 6;28:1. doi: 10.1186/s11658-022-00409-6 (PMC9825046; doi:10.1186/s11658-022-00409-6)
Supplement: Supplementary file 2 — Additional file 2: Figure S1. Summary of somatic mutations and distribution of somatic mutations in ATC and PTC cell lines. Figure S2. Hi-C interaction heat-maps in ATC and PTC cell lines. Figure S3. Motifs analysis for TAD regions with CoMut. Figure S4. TAD A/B compartment switches in ATC cells ordered in each chromosome. [file 11658_2022_409_MOESM2_ESM.pptx]

## Slide 1
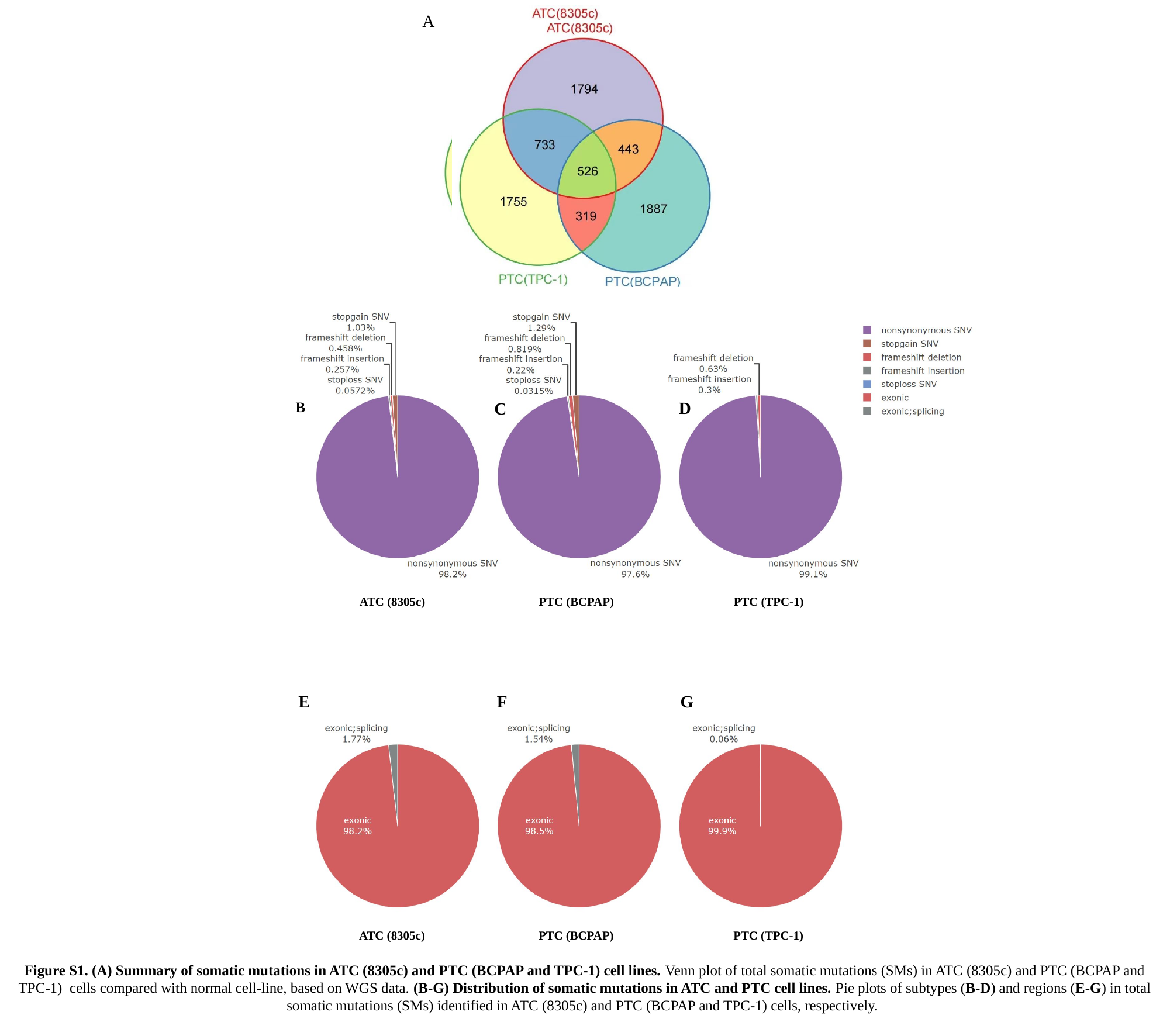

A
D
B
C
E
F
G
 ATC (8305c) PTC (BCPAP) PTC (TPC-1)
 ATC (8305c) PTC (BCPAP) PTC (TPC-1)
Figure S1. (A) Summary of somatic mutations in ATC (8305c) and PTC (BCPAP and TPC-1) cell lines. Venn plot of total somatic mutations (SMs) in ATC (8305c) and PTC (BCPAP and TPC-1) cells compared with normal cell-line, based on WGS data. (B-G) Distribution of somatic mutations in ATC and PTC cell lines. Pie plots of subtypes (B-D) and regions (E-G) in total somatic mutations (SMs) identified in ATC (8305c) and PTC (BCPAP and TPC-1) cells, respectively.

## Slide 2
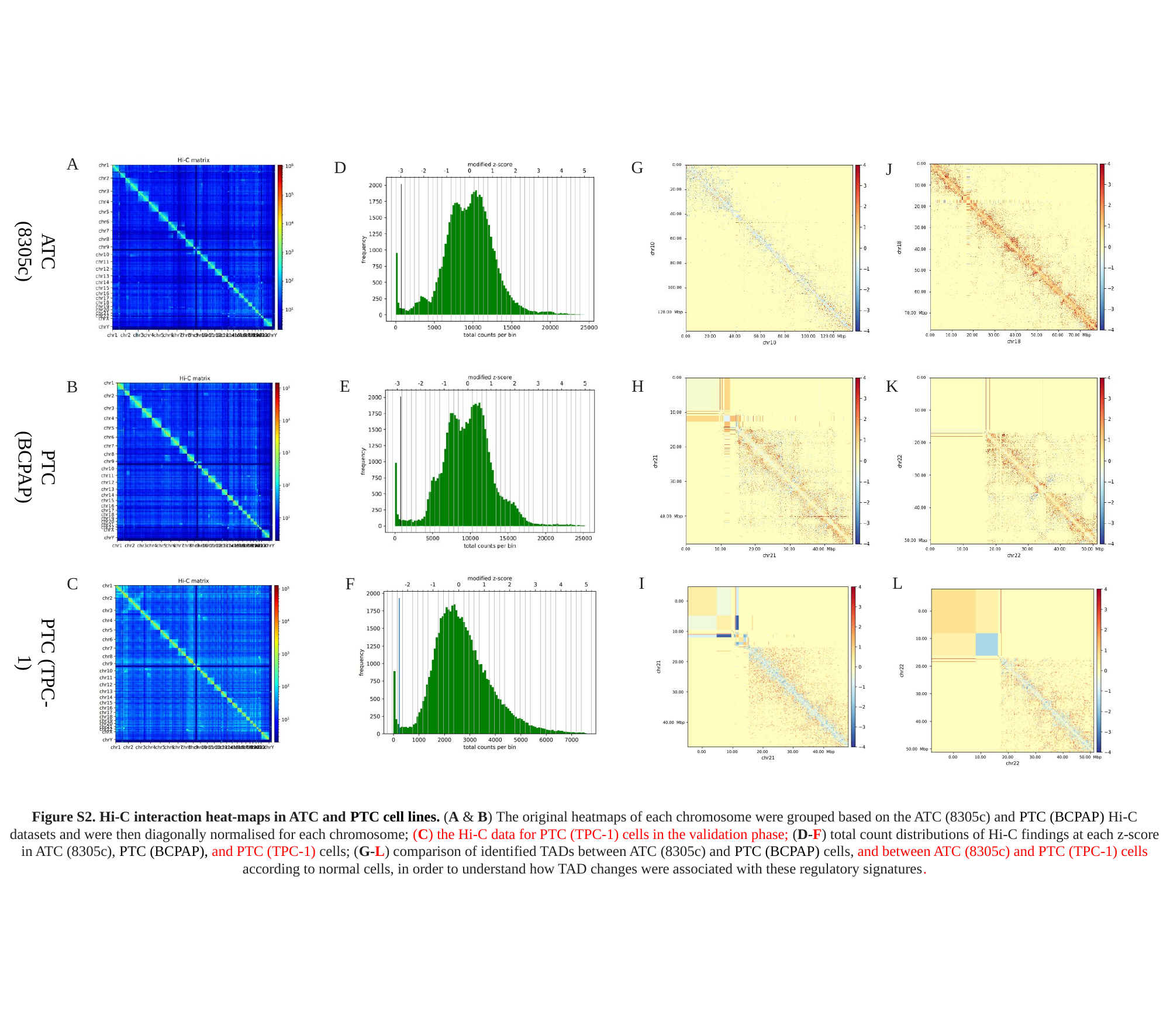

A
D
G
J
E
H
K
B
I
L
C
F
ATC (8305c)
PTC (BCPAP)
PTC (TPC-1)
Figure S2. Hi-C interaction heat-maps in ATC and PTC cell lines. (A & B) The original heatmaps of each chromosome were grouped based on the ATC (8305c) and PTC (BCPAP) Hi-C datasets and were then diagonally normalised for each chromosome; (C) the Hi-C data for PTC (TPC-1) cells in the validation phase; (D-F) total count distributions of Hi-C findings at each z-score in ATC (8305c), PTC (BCPAP), and PTC (TPC-1) cells; (G-L) comparison of identified TADs between ATC (8305c) and PTC (BCPAP) cells, and between ATC (8305c) and PTC (TPC-1) cells according to normal cells, in order to understand how TAD changes were associated with these regulatory signatures.

## Slide 3
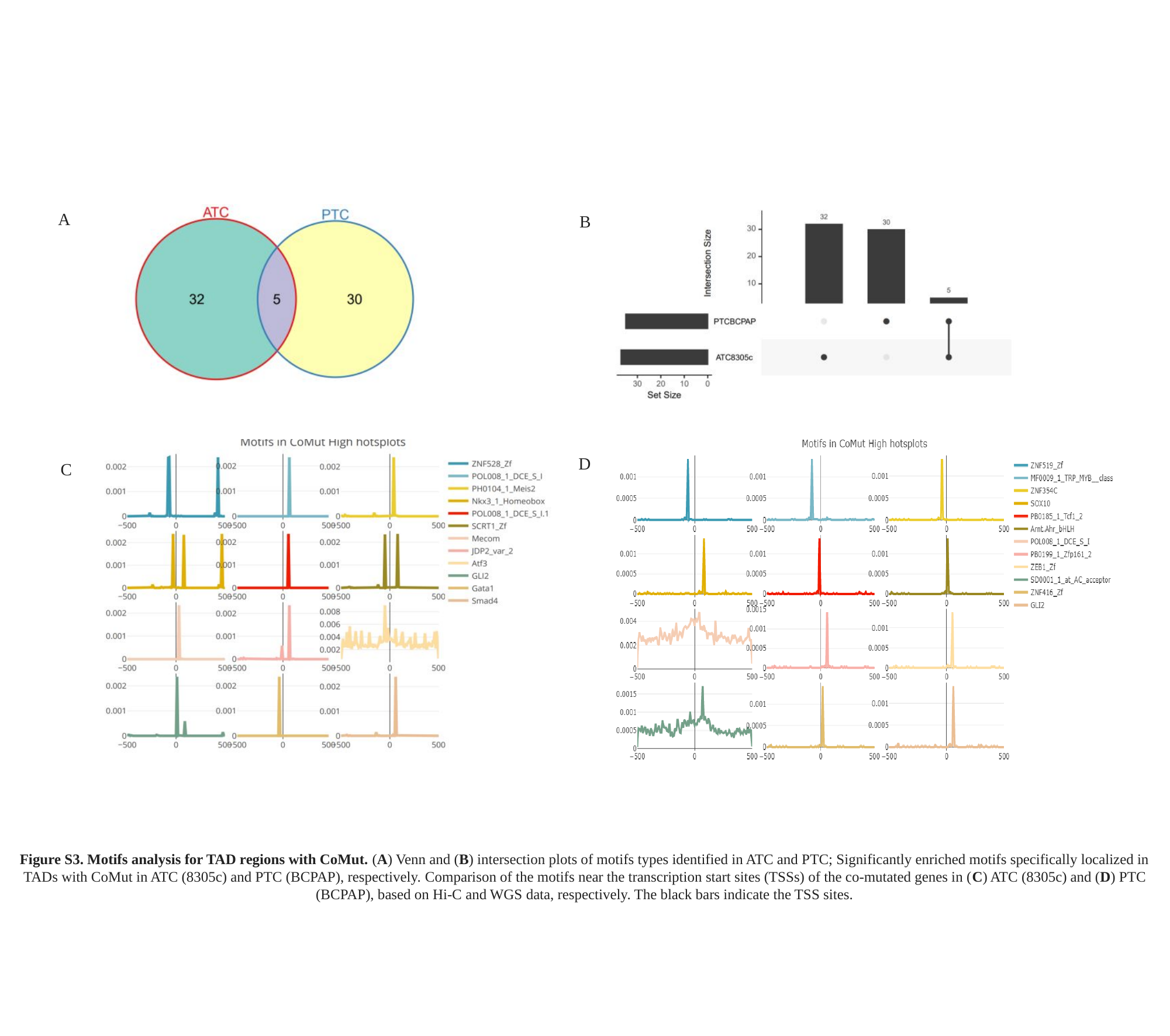

A
B
D
C
Figure S3. Motifs analysis for TAD regions with CoMut. (A) Venn and (B) intersection plots of motifs types identified in ATC and PTC; Significantly enriched motifs specifically localized in TADs with CoMut in ATC (8305c) and PTC (BCPAP), respectively. Comparison of the motifs near the transcription start sites (TSSs) of the co-mutated genes in (C) ATC (8305c) and (D) PTC (BCPAP), based on Hi-C and WGS data, respectively. The black bars indicate the TSS sites.

## Slide 4
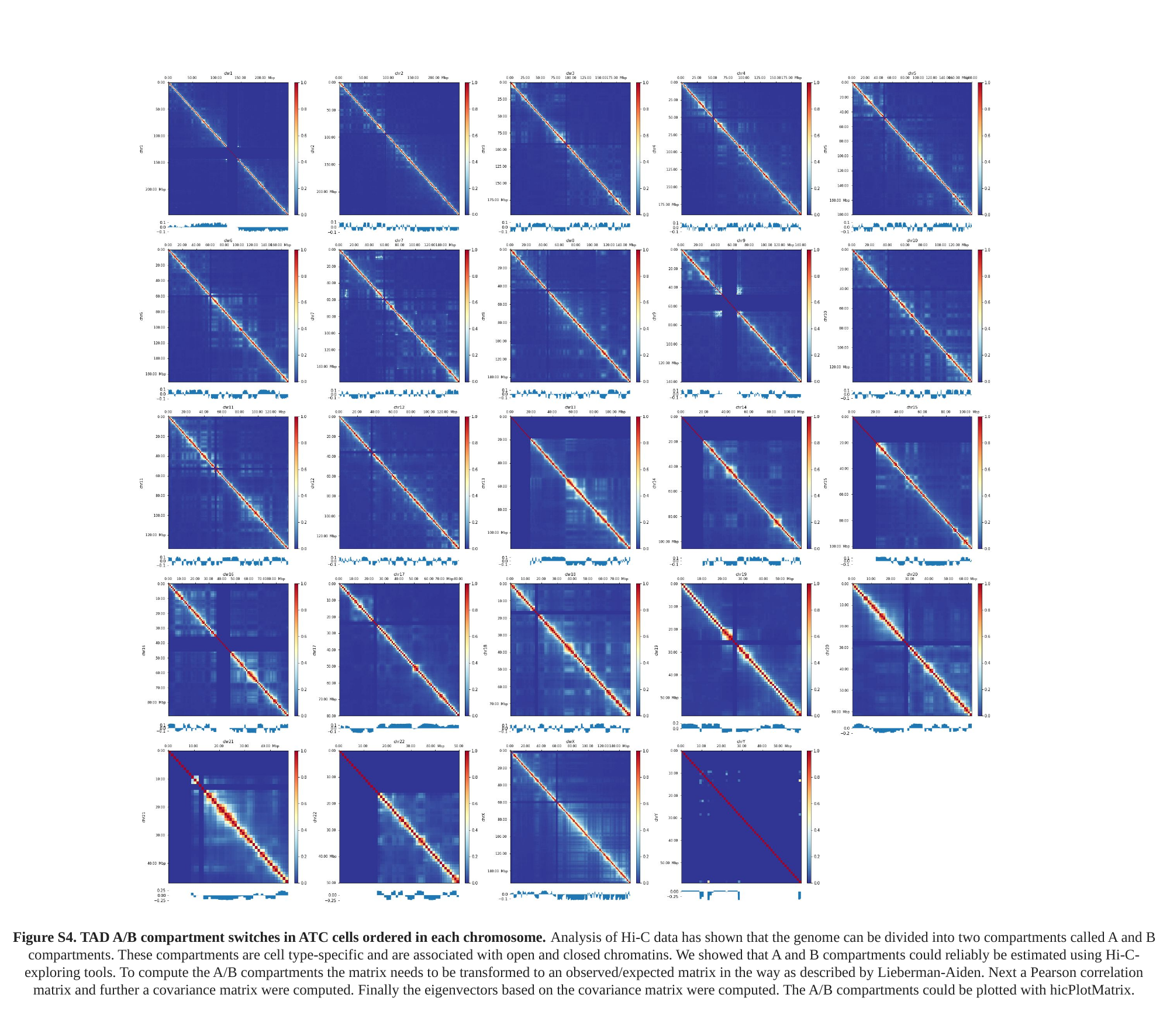

Figure S4. TAD A/B compartment switches in ATC cells ordered in each chromosome. Analysis of Hi-C data has shown that the genome can be divided into two compartments called A and B compartments. These compartments are cell type-specific and are associated with open and closed chromatins. We showed that A and B compartments could reliably be estimated using Hi-C-exploring tools. To compute the A/B compartments the matrix needs to be transformed to an observed/expected matrix in the way as described by Lieberman-Aiden. Next a Pearson correlation matrix and further a covariance matrix were computed. Finally the eigenvectors based on the covariance matrix were computed. The A/B compartments could be plotted with hicPlotMatrix.
